# Copy the In-group: Group Membership Trumps Perceived Reliability, Warmth, and Competence in a Social-Learning Task

**DOI:** 10.1177/09567976211032224

**Published:** 2021-12-23

**Authors:** Marcel Montrey, Thomas R. Shultz

**Affiliations:** 1 Department of Psychology, McGill University; 2 School of Computer Science, McGill University

**Keywords:** cumulative cultural evolution, intergroup dynamics, social-learning strategies, transmission biases, open data, preregistered

## Abstract

Surprisingly little is known about how social groups influence social learning. Although several studies have shown that people prefer to copy in-group members, these studies have failed to resolve whether group membership genuinely affects who is copied or whether group membership merely correlates with other known factors, such as similarity and familiarity. Using the minimal-group paradigm, we disentangled these effects in an online social-learning game. In a sample of 540 adults, we found a robust in-group-copying bias that (a) was bolstered by a preference for observing in-group members; (b) overrode perceived reliability, warmth, and competence; (c) grew stronger when social information was scarce; and (d) even caused cultural divergence between intermixed groups. These results suggest that people genuinely employ a copy-the-in-group social-learning strategy, which could help explain how inefficient behaviors spread through social learning and how humans maintain the cultural diversity needed for cumulative cultural evolution.

As humans, we rely heavily on social learning, which allows us to benefit from others’ knowledge without incurring the same costs or undertaking the same risks (Montrey & Shultz, 2020). However, copying is not always adaptive (Kendal et al., 2018). Not only is there no guarantee that any given individual’s behavior is worth adopting (Rieucau & Giraldeau, 2011), but also changes in the social, ecological, or physical environment often invalidate existing knowledge (Enquist & Ghirlanda, 2007). A mounting body of evidence suggests that social learners avoid such pitfalls by employing various social-learning strategies to determine whom and when to copy (Kendal et al., 2018). For example, by preferentially copying the majority, a migrating individual can adopt locally adaptive behavior while minimizing the risk of copying a fellow migrant (Shultz et al., 2017). Similarly, by prioritizing successful individuals, a learner can be more certain that copied behavior is effective (Sarin & Dukas, 2009). Although a wide range of social-learning strategies have been identified in humans, including biases toward copying older (Wood et al., 2012), socially dominant (Flynn & Whiten, 2012), prestigious (Chudek et al., 2012), and popular (Flynn & Whiten, 2012) individuals, what is less well understood is how social groups affect copying. In particular, are we more likely to copy members of our own group? If so, how might this shape our culture and the course of its evolution?

On its face, an in-group bias in social learning may seem redundant. Because we are often surrounded by in-group members, we would presumably learn our group’s norms and traditions with or without it (Buttelmann et al., 2013). Nevertheless, developmental studies have found that children are more likely to learn (Pető et al., 2018), endorse (Kinzler et al., 2011), and generalize (Oláh et al., 2016) behavior when it is demonstrated by members of their own linguistic group. In certain contexts, this tendency may even extend to infants (Buttelmann et al., 2013; Howard et al., 2015). Likewise, experiments with adults have shown that race can influence social learning of fear and safety (Golkar et al., 2015) and that groups may even affect our willingness to adopt shared linguistic conventions (Shteynberg & Apfelbaum, 2013).

Although these studies paint a clear pattern of behavior, they shed little light on how it arises. There are at least three possible explanations. First, group membership could directly affect social learning. That is, some individuals could be copied more readily solely by virtue of belonging to the same social group. Second, group membership could indirectly affect social learning—for example, because in-group members draw more attention or are perceived as more reliable (Kinzler et al., 2011). Notably, people preferentially attend to in-group members from a very young age (Kinzler et al., 2007) and tend to evaluate them more favorably (Brewer, 1979). Third, group membership could have no effect on social learning at all. Although this may seem counterintuitive, recent findings suggest that people deploy social-learning strategies flexibly and in tandem (Kendal et al., 2018). An apparent in-group-copying bias could in reality consist of a combination of other strategies. For example, conformity bias could mimic an in-group bias when the in-group forms a majority. Likewise, similarity or familiarity bias could underlie a preference for copying in-group members when individuals resemble their own group or have more experience with its appearance or behavior (Buttelmann et al., 2013; Howard et al., 2015; Kinzler et al., 2011). Crucially, people prioritize learning from familiar individuals (Corriveau & Harris, 2009), and even infants prefer to learn from similarly acting others (Gerson et al., 2017). Because studies often manipulate group membership through language (Buttelmann et al., 2013; Howard et al., 2015; Oláh et al., 2016; Pető et al., 2018), accent (Kinzler et al., 2011), or race (Essa et al., 2019; Golkar et al., 2015), such confounding factors are difficult to discount.

One way to isolate the role of group membership is through the minimal-group paradigm, in which novel groups are created and membership is assigned arbitrarily (Diehl, 1990). This prevents groups from having any shared history or unifying features beyond membership and ensures that participants have no personal stake in their group’s success or reputation. Surprisingly, meta-analysis reveals that such groups often elicit in-group favoritism on par with that found in preexisting groups (Balliet et al., 2014). Group membership itself can thus have a remarkably strong effect on behavior.

**Statement of Relevance**

Do people prefer to copy members of their own social group (e.g., people of the same political affiliation, religion, race, etc.), even when they have nothing in common? In this research, we assigned people to random groups and then examined whether this affected who was copied. We found that despite these groups being completely arbitrary and virtually identical, most people preferred to copy their own group’s members. Surprisingly, people who rated their group as less competent were still more likely to copy their fellow group members’ behavior, and this bias even caused the groups’ behavior to measurably diverge. These findings help explain how humans create and maintain cultural diversity, a key driver of technological evolution. They also shed light on why social media seems particularly susceptible to spreading misinformation, fake news, and opposition to vaccination: By segregating people into like-minded communities, these platforms may drive people to copy others more readily—and to do so uncritically.

Here, we disentangled group membership from confounding factors such as similarity and familiarity by randomly assigning participants to two groups. Participants then played a simple game in which social learning could guide their decisions. In Experiment 1, we tested whether group membership affected who was copied and then assessed whether any bias in copying could be attributed to a bias in attention or to a tendency to view in-group members as more reliable. We found a robust in-group-copying bias, which persisted after controlling for attention and arose even among participants who denied that the in-group was more reliable. This bias even caused cultural divergence despite the groups being fully intermixed. In Experiment 2, we built on these findings by varying the amount of social information and by examining participants’ stereotypes about each group. We found that the in-group-copying bias grew with the scarcity of social information and arose even among participants who viewed the out-group as warmer or more competent.

Full data from Experiments 1 and 2 as well as R code for all statistical analyses can be found on OSF (https://doi.org/10.17605/OSF.IO/Z6D7J). For additional analyses and results, see the Supplemental Material available online.

## Experiment 1

### Method

#### Participants

Participants (57% female; mean age = 36.96 years, *SD* = 11.79) were 360 U.S. residents recruited via Amazon Mechanical Turk (MTurk) to play a short game (mean duration = 3.16 min). The first 36 participants had no opportunity for social learning and were thus excluded from our analysis, resulting in a final sample size of 324. Meta-analysis suggests that effect sizes ranging from small to medium are typical of in-group favoritism in experimental games (Balliet et al., 2014). Power analysis suggests that a 95% probability of detecting an effect of this size (Cohen’s *d* = 0.20) requires a sample size of 327 in a within-subjects design. Our final sample size was as close to this value as our experimental design allowed. Participants were paid between $0.10 and $0.30 on the basis of their final score out of 100. This experiment was approved by the McGill University Research Ethics Board.

#### Procedure

After consenting to participate and answering several demographic questions, each participant was randomly assigned to one of two groups. Participants were told their affiliation but not how the groups were formed. Group membership was color coded, but which color was associated with which group varied randomly across participants. Each participant was also assigned to one of six transmission chains through which social information was passed from one experimental generation to the next. This made reciprocal interactions impossible, which prevented social learning from being used to pursue unrelated social goals (Over & Carpenter, 2012) and allowed us to examine cultural change over time (Caldwell et al., 2020). Each generation consisted of three participants from each group (six total). Participants were not told which generation or chain they belonged to.

Next, we provided instructions on how to play “Where’s the Rabbit?” This game has previously been used to study the cost/benefit trade-offs of social and individual learning (Kameda & Nakanishi, 2002) and is similar to a multiarmed bandit. In this game, a simulated rabbit chooses one of two nests to inhabit (A or B), and participants receive 5 points whenever they guess the correct nest. Between rounds, the rabbit has a 90% probability of staying in the same nest and a 10% probability of switching. There are 20 rounds in total. After every five rounds, participants are shown their cumulative score and how it compares with those in the previous generation. This gives participants a sense of their overall performance while avoiding direct feedback about which guesses are correct or which group is more successful. Both before and after the game, participants compared their group’s perceived reliability with that of the other group (*more reliable*, *less reliable*, *equally reliable*, or *not sure/don’t know*).

**Fig. 1.**
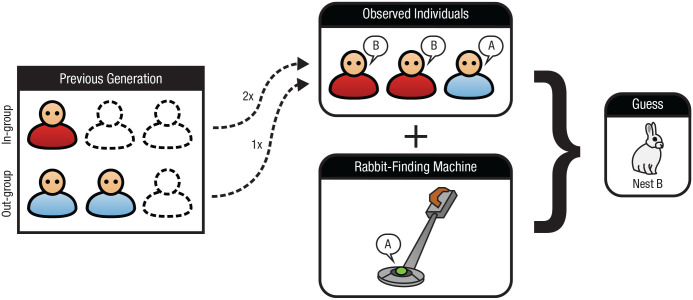
Schematic diagram of a typical round in the “Where’s the Rabbit?” game. In this game, a simulated rabbit chooses one of two nests to inhabit (A or B), and participants receive 5 points whenever they guess the correct nest. Each round began with participants choosing three members of the previous generation to observe. Participants sampled without replacement from the in-group, the out-group, or some combination of the two. They could also use a rabbit-finding machine, which was costly and unreliable. After seeing which nest the three observed individuals and the machine selected, participants guessed the rabbit’s location. In this example, the participant observed two in-group members and one out-group member and opted to use the machine. By guessing nest B, the participant agreed with both in-group members but not the out-group member.

Each round began with participants choosing three members of the previous generation to observe. This involved sampling without replacement from the in-group, the out-group, or some combination of the two. The groups’ left-to-right order was randomized across participants. Participants then decided whether to pay 2 points to use a rabbit-finding machine, which they were told had a two-thirds chance of revealing the correct nest and a one-third chance of giving faulty information. By making this machine costly and unreliable, we allowed participants to glean some direct knowledge about the rabbit’s behavior while still promoting reliance on social learning. Once participants had decided whom to observe and whether to use the machine, they were shown which nest the three observed individuals selected (social learning) and the machine’s results (individual learning). Participants then guessed where the rabbit was and proceeded to the next round. Figure 1 shows a schematic diagram of a typical round.

Participants in the first generation had no one to observe and had to rely on individual learning. Their role was merely to seed each transmission chain with social information. Such information is valuable, because although the rabbit-finding machine’s feedback varied across participants, the rabbit’s actual movements were identical for everyone in the same chain.

#### Measures

##### Copying biases

We measured intergroup biases in copying by comparing how often participants agreed with in-group versus out-group members. Copying was considered biased toward whichever group participants agreed with more often or unbiased if they agreed equally with each group.

##### Attentional biases

We measured intergroup biases in attention by comparing how often participants observed in-group versus out-group members. Attention was considered biased toward whichever group was observed more often or unbiased if the groups were observed at equal rates.

##### Cultural divergence

To measure cultural divergence, we first encoded participants’ guesses as strings of 20 “A” and “B” values. We then counted the differences (Hamming distance) between each string and the strings produced by the previous generation. Cultural divergence occurred when the average Hamming distance was greater from out-group members than from in-group members.

#### Analysis

To test whether participants were more likely to observe or agree with in-group members, we fitted a series of generalized linear models. Participants’ behavior toward each group was treated as a separate within-subjects measure, with group membership (in-group vs. out-group) acting as an independent variable. Each observation or agreement was a dichotomous outcome in that it was either paired with the group in question (success) or not (failure), which we modeled using logistic regression. Because there were exactly two groups, in-group and out-group outcomes were perfectly negatively correlated. We used generalized estimating equations (GEEs) to control for this nonindependence, which allowed us to impose a fixed correlational structure, $\left[\begin{array}{ll} 1 & -1\\ -1 & 1 \end{array}\right]$ . Because estimating equations are not likelihood equations, hypothesis testing in GEE is performed via Wald tests. Single-parameter tests take the form of *z* tests, whereas omnibus tests take the form of χ^2^ tests. We report effect sizes using odds ratios (*OR*s) and McKelvey-Zavoina pseudo-*R*^2^ values, respectively.

### Results

**Fig. 2.**
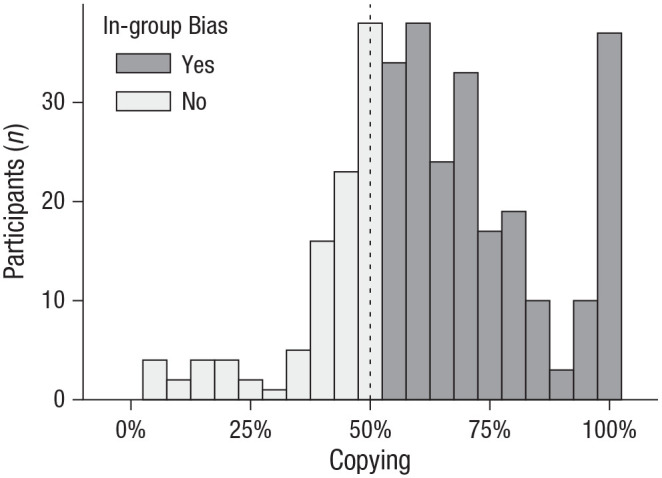
Frequency with which participants copied in-group versus out-group members (Experiment 1). The dotted line represents the expected mean in the absence of an intergroup bias.

Participants showed an in-group bias in copying (Fig. 2). On average, 62% of agreements (95% confidence interval [CI] = [60%, 64%]) were with in-group members, whereas only 38% (95% CI = [36%, 40%]) were with out-group members, *z* = 9.65, p<.001 , *OR* = 2.66 2.66 , 95% CI = [2.18, 3.24].

**Fig. 3.**
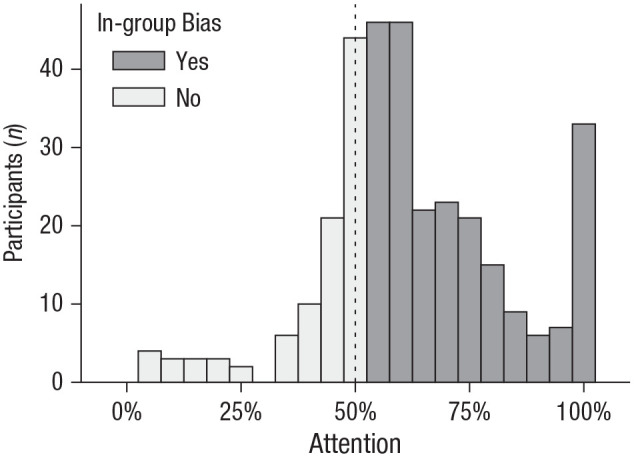
Frequency with which participants observed in-group versus out-group members (Experiment 1). The dotted line represents the expected mean in the absence of an intergroup bias.

Likewise, participants showed an in-group bias in attention (Fig. 3). On average, they observed in-group members 61% of the time, 95% CI = [59%, 64%], but out-group members only 39% of the time, 95% CI = [37%, 41%], *z* = 9.52, p<.001 , OR=2.50 , 95% CI = [2.07, 3.02]. Is the in-group-copying bias driven entirely by differential attention? Controlling for how much more often the in-group was observed, and even how the groups were perceived, failed to eliminate the in-group-copying bias (see “Direct vs. Indirect Bias” in Supplemental Analyses of Copying, which can be found in the Supplemental Material). This suggests that, although much of the copying bias was driven by indirect factors, such as attention, group membership had some direct effect as well.

**Fig. 4.**
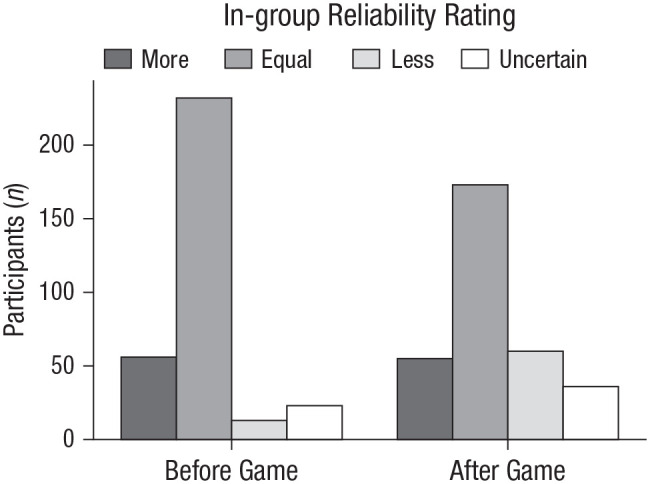
Distribution of in-group reliability ratings before and after the game (Experiment 1). Participants were asked to rate whether their in-group was more reliable, equally reliable, or less reliable compared with the out-group or whether they were uncertain.

The in-group enjoyed a transient advantage in perceived reliability (Fig. 4). Before the game, 17% of participants rated the in-group as more reliable than the out-group, 72% rated the groups as equally reliable, 4% rated the in-group as less reliable than the out-group, and 7% were uncertain. After the game, 17% of participants rated the in-group as more reliable, 53% rated the groups as equally reliable, 19% rated the in-group as less reliable, and 11% were uncertain. Most participants thus explicitly rejected the notion that the in-group was more reliable, both before the game ( M=76% , binomial test: p<.001) , and after it ( M=72% , binomial test: p<.001 ). Of the participants who believed that one group was more reliable, most initially endorsed the in-group ( M=81% , binomial test: p<.001) , though this evened out by the end of the game ( M=48% , binomial test: p=.709) . Perceived reliability had surprisingly little effect on copying; even participants who explicitly rejected the notion that the in-group was more reliable showed an in-group-copying bias (see “Reliability and Copying” in the Supplemental Analyses of Copying).

**Fig. 5.**
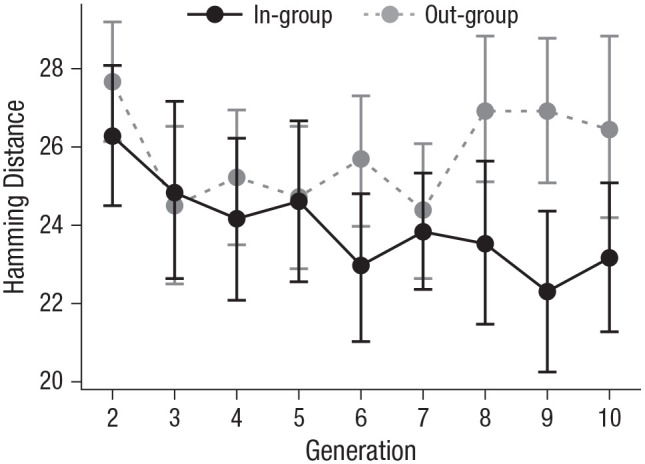
Participants’ average Hamming distance from the in-group and out-group over experimental generations (Experiment 1). Error bars represent bootstrapped 95% confidence intervals.

Despite participants having equal access to both groups, the in-group-copying bias caused cultural divergence. Participants’ average Hamming distance was only 23.97 from the in-group but 25.83 from the out-group, a mean difference of −1.86 (95% CI = [−2.60, −1.13]), *t*(323) = −5.01, *p* < .001, *d* = −0.28, 95% CI = [−0.39, −0.17]. Regressing cultural divergence on experimental generation revealed that the amount of cultural divergence grew over time (Fig. 5), *t*(322) = −3.23, *p* = .001, *r*^2^ = .03, 90% CI = [.01, .07].

## Experiment 2

One possible explanation for the in-group biases in copying and attention is that participants repeatedly observed an odd number of individuals. This forced them to overrepresent either the in-group or the out-group within rounds, which could elicit a preference for in-group members. We tested this hypothesis in Experiment 2 by varying the number of observed individuals.

We also took this opportunity to more closely examine how the groups were perceived. In Experiment 1, participants compared groups directly, which may not fully capture their beliefs. For example, participants could view the in-group more favorably yet be unwilling to explicitly endorse this view in a head-to-head comparison. Therefore, in Experiment 2, participants evaluated each group independently using a validated stereotype questionnaire.

### Method

#### Participants

We recruited 240 new participants (48% female; mean age = 35.93 years, *SD* = 10.15) via MTurk. To ensure that participants were U.S. residents, we used an Internet protocol (IP) look-up service (IPHub) to proactively filter anyone connecting from a non-U.S. IP address or a virtual private server. As in Experiment 1, the first generation was excluded from analysis, resulting in a final sample size of 216. Also, one participant failed to contribute any copying data because they never agreed with either group. In determining our sample size, we aimed to reliably detect the presence or absence of both the in-group-copying bias and the in-group attentional bias in each condition. To estimate this value, we ignored distributional assumptions and used a paired-samples *t* test to approximate the effect size of the weaker bias, which is the bias in attention (*d* = 0.55). Power analysis suggested that 45 participants were needed for a 95% chance of detecting an effect of this size. Recruiting one chain of 54 participants per condition brought us as close to this value as our experimental design allowed. The payment scheme was identical to Experiment 1’s, and mean game duration (3.81 min) was similar. This experiment was approved by the McGill University Research Ethics Board.

#### Procedure

Experiment 2 followed the same design as Experiment 1, with two notable exceptions. First, participants were randomly assigned to different conditions, where they observed either one, two, three, or four individuals. Second, participants rated each group independently using a validated stereotype-content-model questionnaire, in which warmth and competence were each measured using a two-item scale (Cuddy et al., 2007). Participants were asked four questions about each group, both before and after the game (16 total). These questions were, “If you had to guess . . . How [competent/capable/warm/friendly] is [your group/the other group]?” Participants responded using a Likert scale ranging from 1 (*not at all*) to 5 (*extremely*), and their responses were averaged across related items. Scale reliability was consistent with results found in previous studies (Cuddy et al., 2007)—warmth: α=.77 , 95% CI = [.73, .80]; competence: α=.81 , 95% CI = [.78, .84]. We randomized question order across participants as well as the order in which we asked about each group.

Our preregistered hypotheses, experimental design, sampling plan, variables, and analysis plan can be found on OSF (https://doi.org/10.17605/OSF.IO/Z6D7J). Notable deviations are explained in the “Preregistration” section of Supplemental Information About Data Quality, Preregistration, and Other Results.

#### Measures

To measure intergroup perceptual biases, we subtracted participants’ out-group warmth or competence ratings from their in-group warmth or competence ratings. Positive values indicated that the in-group was seen as warmer or more competent, negative values indicated that the out-group was seen as warmer or more competent, and zero indicated that the groups were seen as equally warm or competent.

### Results

**Fig. 6.**
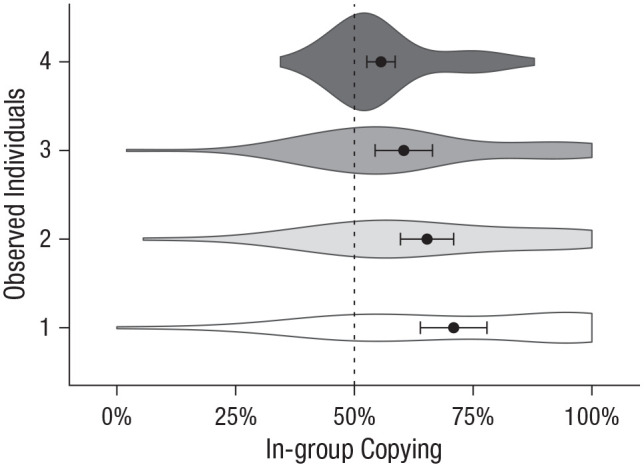
Frequency with which participants copied in-group versus out-group members as a function of the number of observed individuals (Experiment 2). Circles represent means, error bars represent 95% confidence intervals, and the height and width of the distributions indicate the density and range, respectively, of the data. The dotted line represents expected means in the absence of an intergroup bias.

The in-group-copying bias varied with the number of observed individuals (Fig. 6), Wald $\chi^{2}(3)=19.30$ , p<.001 , partial $R^{2}=.05$ , 90% CI = [.02, .08]. According to Bonferroni-corrected post hoc tests, participants who observed four individuals per round showed a weaker in-group-copying bias than those who observed one or two individuals. On average, 71% of agreements (95% CI = [64%, 77%]) were with in-group members when participants observed one individual per round, z=5.18 , p<.001 , OR=5.94 , 95% CI = [3.03, 11.64]; 65% (95% CI = [60%, 71%]) were with in-group members when they observed two individuals per round, z=5.02 , p<.001 , OR=3.55 , 95% CI = [2.16, 5.82]; 60% (95% CI = [54%, 66%]) were with in-group members when they observed three individuals per round, z=3.28 , p=.001 , OR=2.32 , 95% CI = [1.40, 3.84]; and 56% were with in-group members (95% CI = [53%, 59%]) when they observed four individuals per round, z=3.65 , p<.001 , OR=1.57 , 95% CI = [1.23, 1.99]. This suggests that the in-group-copying bias was not contingent on observing an odd number of individuals but instead grew with the scarcity of social information. As in Experiment 1, controlling for both attentional and perceptual biases failed to eliminate the in-group-copying bias, which suggests that group membership had some direct effect on copying (see “Direct vs. Indirect Bias” in Supplemental Analyses of Copying).

**Fig. 7.**
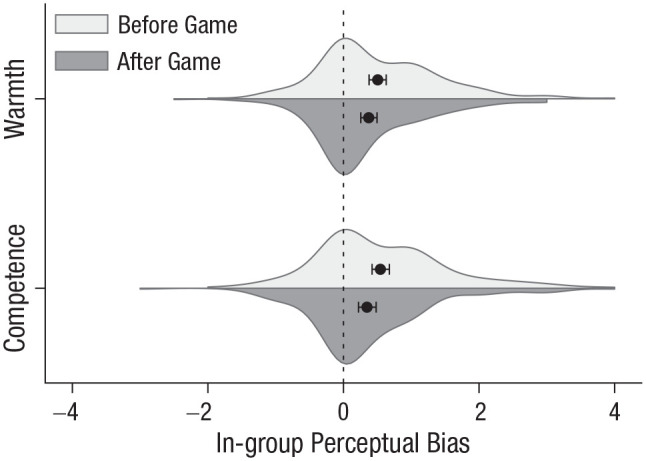
Difference between in-group and out-group ratings of warmth and competence before and after the game (Experiment 2). Circles represent means, error bars represent bootstrapped 95% confidence intervals, and the height and width of the distributions indicate the density and range, respectively, of the data. The dotted line represents expected means in the absence of an intergroup bias.

The in-group was viewed as both warmer and more competent than the out-group (Fig. 7). Before the game, participants rated the in-group an average of 0.51 points (95% CI = [0.39, 0.63]) warmer, t(215)=8.39 , p<.001 , d=0.57 , 95% CI = [0.43, 0.71], and 0.53 points (95% CI = [0.40, 0.66]) more competent, t(215)=8.21 , p<.001 , d=0.56 , 95% CI = [0.41, 0.70]. After the game, participants rated the in-group an average of 0.39 points (95% CI = [0.28, 0.50]) warmer, t(215)=6.77 , p<.001 , d=0.46 , 95% CI = [0.32, 0.60], and 0.36 points (95% CI = [0.24, 0.47]) more competent, t(215)=6.01 , p<.001 , d=0.41 , 95% CI = [0.27, 0.55]. As in Experiment 1, beliefs about the groups had surprisingly little effect on copying; even participants who rated the out-group as warmer or more competent showed an in-group-copying bias (see “Warmth, Competence, and Copying” in Supplemental Analyses of Copying).

The number of observed individuals had no effect on either the amount, F(3,212)=1.78 , p=.152 , $\eta^{2}=.02$ , 90% CI = [.00, .06], or the rate of cultural divergence, F(3,208)=0.60 , p=.617 , η_*p*_^2^ =.00 , 90% CI = [.00, .03]. Across conditions, participants’ average Hamming distance was only 26.37 from the in-group but 27.28 from the out-group, a mean difference of −0.91 (95% CI = [−1.80, −0.01]), t(215)=−1.99 , p=.047 , d=−0.14 , 95% CI = [ −0.27 , −0.00 ]. However, unlike in Experiment 1, the amount of cultural divergence did not grow over time, t(214)=0.39 , p=.694 , $r^{2}=.00$ , 90% CI = [.00, .02]. Given the smaller sample size in Experiment 2, it is possible that this growth was simply harder to detect than the mere presence of cultural divergence. These effects may also have been attenuated because participants observed fewer individuals, on average, than in Experiment 1.

## Discussion

Although previous studies have found an apparent in-group bias in social learning, they have failed to resolve whether this constitutes a genuine social-learning strategy or a mere confluence of other factors (Buttelmann et al., 2013; Howard et al., 2015). Our study disentangled group membership from similarity and familiarity by assigning group membership at random. We found that rather than eliminating the preference for in-group members, this approach resulted in a robust in-group-copying bias, which (a) was bolstered by a tendency to observe in-group members, (b) overrode participants’ stated beliefs, (c) grew stronger when social information was scarce, and (d) even caused cultural divergence between intermixed groups. Taken together, our findings suggest that people genuinely employ a copy-the-in-group strategy and that group membership has both a direct and indirect effect on copying.

Why might a copy-the-in-group strategy have evolved in the first place? One reason could be that it allowed humans to rapidly adopt and vigorously maintain group norms that enhance coordination (McElreath et al., 2003) or promote cooperation (Boyd & Richerson, 2009). Another reason could be that social learning is useful only to the extent that adopting other people’s behavior yields similar payoffs (Laland, 2004). For example, copying out-group members could be less efficient or even counterproductive if groups differ in terms of what behavior is punished or rewarded. Finally, such a strategy could also have evolved because it minimized the risk of deception. Because social learning is essentially information scrounging (Kameda & Nakanishi, 2002), in which the copier benefits from other people’s knowledge without incurring the same costs, knowledgeable individuals have an incentive to mislead others. However, this incentive is minimized when observed individuals have a vested interest in the copier’s success. This holds true in kin relationships (Laland, 2004) and likely generalizes to other settings, such as intergroup competition.

Although the in-group-copying bias may be adaptive, we were careful to control for factors that could justify such a bias or evoke a similar-looking one. First, we limited the role of similarity and familiarity by assigning participants to groups at random. Second, we ensured that there was no inherent advantage to copying either group by giving participants access to the same information and by discouraging deception. Third, we deterred intergroup competition by framing the game in terms of individual performance and by obscuring group membership whenever participants saw others’ scores. Fourth, we prevented selective copying from advancing social goals (Over & Carpenter, 2012), such as inclusion (Watson-Jones et al., 2016), by limiting social interactions to unidirectional observations of previous participants. Finally, we matched everyone with an equal number of participants from each group to ensure that fellow in-group members did not form a majority. That being said, because cultures could conceivably vary in their reliance on group membership, the generalizability of our findings may be limited by our sample consisting solely of U.S. residents. Furthermore, although online recruitment platforms often yield a more diverse pool of participants than traditional student samples, they raise other concerns about data quality. For a brief overview of these concerns and our analysis of their impact, see “Data Quality” in Supplemental Information About Data Quality, Preregistration, and Other Results.

In addition to controlling for potential confounds, we also tested several proposed explanations for why people may preferentially copy in-group members. One is that we attend to them more often (Kinzler et al., 2011). This seems sensible, given that native-language speakers attract more attention from a very young age (Kinzler et al., 2007) and that adults bias their attention toward in-group members, whether that group is preexisting or novel (Kawakami et al., 2014). However, developmental studies have failed to reveal any obvious in-group attentional bias in social learning (Oláh et al., 2016), and both looking times (Buttelmann et al., 2013; Pető et al., 2018) and eye-tracking data (Howard et al., 2015) contradict this hypothesis. It is therefore curious that most participants in our experiments (70% and 67%) observed the in-group more often and that this drove much of the in-group bias in copying. One possibility is that humans become more likely to attend to in-group members over the course of development. For example, instead of attention guiding children’s copying, biases in copying could shape their attention. Another possibility is that attention played a greater role in our study because participants explicitly chose whom to observe and paid a clear opportunity cost for each selection. Our findings may thus be particularly relevant to contexts in which people have strong and explicit control over their sources of social information, such as on social media.

Another proposed explanation for why people may prefer to copy in-group members is that we ascribe greater competence to the in-group (Kinzler et al., 2011) or view out-group members as less reliable (Oláh et al., 2016). This hypothesis is rooted in the classic social-psychology finding that in-group members are evaluated more favorably, even when groups are arbitrary and novel (Brewer, 1979). Indeed, we found that the in-group enjoyed advantages in perceived reliability, warmth, and competence. However, none of these beliefs had much effect on whom participants copied. On the contrary, because the in-group-copying bias arose even among participants who viewed the out-group as more competent, our results seemingly contradict the axiom that social-learning strategies exist solely to help people identify and adopt the most effective behavior (Kendal et al., 2018).

In fact, if people are predisposed to copy in-group members, perhaps even when their perceived competence is low, this could help explain the spread of inefficient or even deleterious behaviors. For example, opposition to vaccination is often disseminated through highly clustered and enclosed online communities (Yuan & Crooks, 2018) who use in-group-focused language (Mitra et al., 2016). Likewise, fake news tends to spread among politically aligned individuals (Grinberg et al., 2019), and the most effective puppet accounts prefer to portray themselves as in-group members rather than as knowledgeable experts (Xia et al., 2019). Our research also sheds light on why social media platforms seem especially prone to spreading misinformation. By offering such fine-grained control over whom users observe, these platforms may spur the creation of homogeneous social networks, in which individuals are more inclined to copy others because they belong to the same social group.

Finally, the fact that the in-group-copying bias produced some amount of cultural divergence in both of our experiments is of particular interest from a cultural evolutionary point of view. This is because the exceptional complexity of human culture and technology (Montrey & Shultz, 2020) likely depends on integrating and recombining diverse cultural traits (Mesoudi & Thornton, 2018). Theory suggests that for cultural evolution to be cumulative (i.e., for complexity to increase over time), populations may have to be fragmented to some degree so that unique traits have the opportunity to flourish (Derex et al., 2018). Otherwise, cultural traits may homogenize, leaving learners with little to recombine. Although the intergroup differences in behavior we observed do not rise to the level of cultural traditions, our findings could help explain how cultural differences persist in intermixed groups (McElreath, 2004). A copy-the-in-group strategy could thus be one mechanism for achieving the cultural diversity needed for cumulative cultural evolution.

## Supplemental Material

sj-pdf-1-pss-10.1177_09567976211032224 – Supplemental material for Copy the In-group: Group Membership Trumps Perceived Reliability, Warmth, and Competence in a Social-Learning Task Supplemental material, sj-pdf-1-pss-10.1177_09567976211032224 for Copy the In-group: Group Membership Trumps Perceived Reliability, Warmth, and Competence in a Social-Learning Task by Marcel Montrey and Thomas R. Shultz in Psychological Science

sj-pdf-2-pss-10.1177_09567976211032224 – Supplemental material for Copy the In-group: Group Membership Trumps Perceived Reliability, Warmth, and Competence in a Social-Learning Task Supplemental material, sj-pdf-2-pss-10.1177_09567976211032224 for Copy the In-group: Group Membership Trumps Perceived Reliability, Warmth, and Competence in a Social-Learning Task by Marcel Montrey and Thomas R. Shultz in Psychological Science

## Transparency

*Action Editor:* Mark Brandt

*Editor:* Patricia J. Bauer

*Author Contributions*

M. Montrey developed the study concept and design, collected and analyzed the data, and wrote the manuscript under the supervision of T. R. Shultz, who helped with comments, suggestions, and writing. Both authors approved the final version of the manuscript for submission.

**Open Practices:** All data and analysis code have been made publicly available via OSF and can be accessed at https://doi.org/10.17605/OSF.IO/Z6D7J. The design and analysis plans for Experiment 2 were preregistered at https://osf.io/wqd8n/. Deviations from the preregistration are discussed in the “Preregistration” section of Supplemental Information About Data Quality, Preregistration, and Other Results, which can be found in the Supplemental Material available online. This article has received the badges for Open Data and Preregistration. More information about the Open Practices badges can be found at http://www.psychologicalscience.org/publications/badges.
