# Supplementary material for: Copy the In-group: Group Membership Trumps Perceived Reliability, Warmth, and Competence in a Social-Learning Task
Source: Psychol Sci. 2021 Dec 23;33(1):165–74. doi: 10.1177/09567976211032224 (PMC13038129; doi:10.1177/09567976211032224)
Supplement: sj-pdf-1-pss-10.1177_09567976211032224 – Supplemental material for Copy the In-group: Group Membership Trumps Perceived Reliability, Warmth, and Competence in a Social-Learning Task [file sj-pdf-1-pss-10.1177_09567976211032224.pdf]

Supplemental Analyses of Copying

Marcel Montrey[1] and Thomas R. Shultz[1,2]

[1]Department of Psychology, McGill University, Montreal, Quebec, Canada
[2]School of Computer Science, McGill University, Montreal, Quebec, Canada

Direct vs. Indirect Bias

To test whether group membership had a direct effect on copying, we first controlled for attention. We did so by counting how many opportunities participants had to agree with each group and then comparing how likely they were to capitalize on those opportunities. For example, observing two members of a group provided two opportunities to agree with that group—unless they disagreed with each other, in which case it provided only one. On this measure, an in-group-copying bias only existed if participants copied in-group members in excess of what was expected given how often the in-group was observed. Because this measure partially decoupled in-group and out-group copying rates, we fitted an exchangeable correlational structure. After controlling for attention, participants were still more likely to copy in-group members—experiment 1: $Z = 4.94$, $p < .001$, $OR = 1.34$, 95% CI = [1.19, 1.50]; experiment 2: $Z = 3.22$, $p = .001$, $OR = 1.25$, 95% CI = [1.09, 1.44]. This suggests that, although much of the in-group-copying bias may be driven by differential attention, group membership had some direct effect as well.

To further corroborate this finding, we also controlled for how the groups were perceived. In experiment 1, we added two dichotomous variables, which indicated whether a participant's pre-game or post-game ratings favored either group. Similarly, in experiment 2, we added four continuous variables, which captured a participant's pre-game and post-game biases in perceived warmth and competence. For each added variable, we also included a two-way interaction between that variable and group membership. We then estimated marginal means for when there was no perceptual bias. Controlling for both perceptual and attentional biases failed to eliminate the in-group-copying bias—experiment 1: $Z = 4.41$, $p < .001$, $OR = 1.39$, 95% CI = [1.20, 1.61]; experiment 2: $Z = 2.85$, $p = .004$, $OR = 1.26$, 95% CI = [1.07, 1.48]. Although these results cannot conclusively show that group membership had a direct effect on who was copied, they support that possibility.

Reliability and Copying

Pre-game reliability ratings had no effect on copying, Wald $\chi^2(3) = 5.44$, $p = .142$, partial $R^2 = .00$, 90% CI = [.00, .00]. This suggests that the in-group-copying bias was not rooted in viewing the in-group as more reliable. In fact, there was direct evidence of an in-group-copying bias not just among participants who rated the in-group as more reliable, $Z = 4.81$, $p < .001$, $OR = 3.14$, 95% CI = [1.97, 5.02]; but also those who rated the groups as equally reliable, $Z = 7.99$, $p < .001$, $OR = 2.66$, 95% CI = [2.10, 3.39]; and those who were uncertain, $Z = 2.68$, $p = .008$, $OR = 2.51$, 95% CI = [1.28, 4.92]. In other words, explicitly rejecting the notion that the in-group was more reliable did not preclude participants from preferentially copying in-group members. Although we could not directly show that participants who rated the in-group as less reliable preferred to copy in-group members, $Z = 0.90$, $p = .369$, $OR = 1.32$, 95% CI = [0.72, 2.42]; we note that this sample size was small ($n = 13$).

Post-game reliability ratings did reflect differences in copying, Wald $\chi^2(3) = 18.16$, $p < .001$, partial $R^2 = .01$, 90% CI = [.01, .03]. According to Bonferroni-corrected post hoc tests, participants who rated the in-group as more reliable showed a stronger preference for copying in-group members than than those who rated it as equally reliable or less reliable. However, it was not just participants who rated the in-group as more reliable who preferred to copy in-group members, $Z = 6.35$, $p < .001$, $OR = 5.80$, 95% CI = [3.37, 9.97]; but also those who rated the groups as equally reliable, $Z = 7.18$, $p < .001$, $OR = 2.55$, 95% CI = [1.98, 3.30]; and those who were uncertain, $Z = 3.69$, $p < .001$, $OR = 3.16$, 95% CI = [1.72, 5.81]. Once again, we could not directly show that those who rated the in-group as less reliable preferred to copy in-group members, $Z = 1.34$, $p = .179$, $OR = 1.34$, 95% CI = [0.88, 2.05].

Warmth, Competence, and Copying

To test whether pre-game biases in perceived warmth and competence affected copying, we added these as continuous variables, which interacted with group membership. The in-group bias in copying was unaffected by either perceptual bias—warmth: $Z = -0.32$, $p = .746$, $OR = 0.95$, 95% CI = [0.70, 1.29]; competence: $Z = 1.71$, $p = .087$, $OR = 1.28$, 95% CI = [0.97, 1.70]. Remarkably, when we discretized perceptual biases to simply reflect their direction (in-group, out-group, or neither), the in-group-copying bias arose even among participants who viewed the out-group as warmer, $Z = 2.15$, $p = .031$, $OR = 1.84$, 95% CI = [1.06, 3.21]; or more competent, $Z = 2.21$, $p = .027$, $OR = 1.67$, 95% CI = [1.06, 2.63].

We repeated this analysis for post-game perceptual biases. Although biases in perceived warmth did not reflect differences in copying, $Z = -0.67$, $p = .506$, $OR = 0.90$, 95% CI = [0.65, 1.24]; biases in perceived competence did, $Z = 2.42$, $p = .016$, $OR = 1.44$, 95% CI = [1.07, 1.94]. Specifically, participants who rated the in-group as more competent were more likely to copy it. However,

discretizing perceptual biases once again revealed that the in-group-copying bias arose even among participants who viewed the out-group as warmer, $Z = 3.01$, $p = .003$, $OR = 2.11$, 95% CI = [1.30, 3.43]; or more competent, $Z = 2.01$, $p = .045$, $OR = 1.53$, 95% CI = [1.01, 2.32].
