# Supplementary material for: Copy the In-group: Group Membership Trumps Perceived Reliability, Warmth, and Competence in a Social-Learning Task
Source: Psychol Sci. 2021 Dec 23;33(1):165–74. doi: 10.1177/09567976211032224 (PMC13038129; doi:10.1177/09567976211032224)
Supplement: sj-pdf-2-pss-10.1177_09567976211032224 – Supplemental material for Copy the In-group: Group Membership Trumps Perceived Reliability, Warmth, and Competence in a Social-Learning Task [file sj-pdf-2-pss-10.1177_09567976211032224.pdf]

Supplemental Information About Data Quality, Preregistration, and Other Results

Marcel Montrey[1] and Thomas R. Shultz[1,2]

[1]Department of Psychology, McGill University, Montreal, Quebec, Canada
[2]School of Computer Science, McGill University, Montreal, Quebec, Canada

Data Quality

Although MTurk and other online recruitment platforms provide easy and low-cost access to a diverse pool of participants (Coppock, 2019), the quality of the data obtained using such platforms is not always consistent. For example, researchers observed a substantial drop in data quality on MTurk during the summer of 2018 (Chmielewski & Kucker, 2020). Later analysis revealed that this drop likely stemmed from the use of virtual private servers (VPSs) by individuals outside the U.S. to circumvent MTurk's geographical filtering tools (Kennedy et al., 2020). Therefore, one proposed method of diagnosing the impact of "bad actors" is to use an IP lookup service to geographically trace IP addresses and to identify VPS use (Kennedy et al., 2020). Although foreign IP addresses may belong to U.S. residents who are traveling, studying, or working abroad, and the use of a VPS could be similarly innocuous, these factors can be predictive of lower quality data (Dennis et al., 2020).

We used IPHub to analyze the IP addresses of the 324 participants who composed our final sample in experiment 1. The IP addresses of 306 of these participants (94%) originated in the U.S., as expected, while the remaining 18 (6%) originated in a wide range of other countries: two each from Canada, Honduras, India, and Seychelles; and one each from Belize, Chile, Colombia, Egypt, Guyana, Serbia, South Africa, Sweden, the United Kingdom, and Venezuela. However, 36 IP addresses (11%) originated from a VPS. Between geolocation information and VPS use, we flagged 46 participants' (14%) data for further examination.

These 46 participants did not appear to differ from the rest of the sample in terms of their in-group-copying bias, Wald $\chi^2(1) = 0.30$, $p = .585$, partial $R^2 = .00$, 90% CI = [.00, .00]; in-group attentional bias, $\chi^2(1) = 0.09$, $p = .768$, partial $R^2 = .00$, 90% CI = [.00, .00]; pre-game ratings, $\chi^2(3, N = 324) = 7.23$, $p = .065$, Cramér's $V = .15$, 95% CI = [.00, .24]; amount of cultural divergence, $F(1, 322) = 0.89$, $p = .346$, $\eta^2 = .00$, 95% CI = [.00, .02]; or rate of cultural divergence, $F(1, 320) = 0.00$, $p = .964$, partial $\eta^2 = .00$, 95% CI =

[.00, .00]. Although they differed in post-game ratings, $\chi^2(3, N = 324) = 9.15$, $p = .027$, $V = .17$, 95% CI = [.00, .26]; Bonferroni-corrected post hoc tests failed to identify which rating was responsible. Further examination revealed no difference in how these participants' post-game ratings affected their copying, Wald $\chi^2(3) = 0.82$, $p = .846$, partial $R^2 = .00$, 90% CI = [.00, .00]; or their attention, Wald $\chi^2(3) = 0.55$, $p = .908$, partial $R^2 = .00$, 90% CI = [.00, .00]. Moreover, these participants showed the same qualitative patterns in their post-game ratings as the rest of our sample. Most explicitly rejected the notion that the in-group was more reliable, $M = 76\%$, binomial test: $p < .001$; while those who viewed one of the groups as more reliable were evenly split, $M = 38\%$, binomial test: $p = .308$.

In short, these 46 participants' data were not of noticeably lower quality, and their inclusion had no discernible impact on the conclusions we drew from experiment 1. Nevertheless, we took more aggressive measures to ensure data quality in experiment 2. We used IPHub to proactively check the IP address of every potential participant before they joined. If an IP address originated from outside the U.S. or from a VPS, that individual was automatically prevented from participating.

Preregistration

Experiment 2 largely conformed to our preregistration. However, there were several notable deviations, which we explain here.

First, as we noted in our preregistration, measures of in-group copying and attention must be standardized to fall in the same range when the number of observed individuals varies. This requires comparing the proportion of in-group versus out-group agreements or observations, rather than the totals. However, due to numerous participants copying and attending exclusively to the in-group, these data were bimodally rather than normally distributed. The standard parametric models we planned to apply were thus invalid. We instead used logistic regression and GEE, replacing t-tests and ANOVAs with the equivalent single parameter and omnibus Wald tests. We reported effect sizes for omnibus tests using McKelvey and Zavoina's (1975) pseudo-R^2 because this tends to produce values comparable to normal linear models (Veall & Zimmermann, 1996). Confidence intervals for R^2 values were obtained via bootstrapping.

Second, when examining whether the in-group bias in copying was fully accounted for by the in-group bias in attention, we used a more sophisticated approach than the one outlined in our analysis plan. Instead of trying to statistically control for attention, we treated copying data as if they originated from an unbalanced design.

Finally, although we collected the same raw measures as outlined in our preregistration, how we computed the in-group-copying bias changed. Initially, we planned to compare how often participants agreed with each group's majority opinion, which we defined as the nest that most of its members were seen to prefer. From a participant's perspective, majority opinions only ex-

isted on rounds where at least one group member was observed and opinions were unevenly split. Unfortunately, this measure had several drawbacks. As one reviewer noted, majorities are typically defined as consisting of multiple individuals. It is not immediately apparent that a single observed individual constitutes a majority in the same sense. This resulted in inconsistencies across conditions in terms of what kinds of majorities were likely or even possible. For example, when observing one individual, every opinion was a majority opinion, but majorities never consisted of more than one person. Moreover, by failing to distinguish between instances where participants agreed with many individuals and those where they agreed with few or only one, this measure obscured much of the variability in the data. As a result, it likely understated the role of attention and may have produced effect sizes that failed to accurately reflect participants' behavior. To address these concerns, we modified this measure so that its interpretation was more straightforward and remained consistent across both rounds and conditions. Instead of comparing how often participants agreed with each group's majority opinion, we compared how often they agreed with each group's members.

Other Results

Copying and Attention

Because an individual could not be copied unless they were observed, copying and attention were strongly correlated—experiment 1: $t(322) = 31.94$, $p < .001$, $r = .87$, 95% CI = [.84, .90]; experiment 2: $t(214) = 27.91$, $p < .001$, $r = .89$, 95% CI = [.85, .91]. For example, the relatively few participants who preferred to observe out-group members also copied them more often—experiment 1: $Z = -7.24$, $p < .001$, $OR = 0.34$, 95% CI = [0.26, 0.46]; experiment 2: $Z = -3.89$, $p < .001$, $OR = 0.51$, 95% CI = [0.37, 0.72].

Reliability and Attention

Pre-game reliability ratings had no effect on whom participants observed, Wald $\chi^2(3) = 6.62$, $p = .085$, partial $R^2 = .00$, 90% CI = [.00, .00]. In fact, there was direct evidence of an in-group attentional bias not just among participants who rated the in-group as more reliable, $Z = 4.55$, $p < .001$, $OR = 2.89$, 95% CI = [1.83, 4.57]; but also those who rated the groups as equally reliable, $Z = 7.95$, $p < .001$, $OR = 2.53$, 95% CI = [2.01, 3.18]; and those who were uncertain, $Z = 2.58$, $p = .010$, $OR = 2.29$, 95% CI = [1.22, 4.30]. Although we could not directly show that participants who rated the in-group as less reliable preferred to observe in-group members, $Z = 1.00$, $p = .317$, $OR = 1.29$, 95% CI = [0.78, 2.14]; we note that this sample size was small ($n = 13$).

Post-game reliability ratings did reflect differences in attention, Wald $\chi^2(3) = 12.17$, $p = .007$, partial $R^2 = .01$, 90% CI = [.00, .02]. According to Bonferroni-corrected post hoc tests, participants who rated the in-group as more reliable

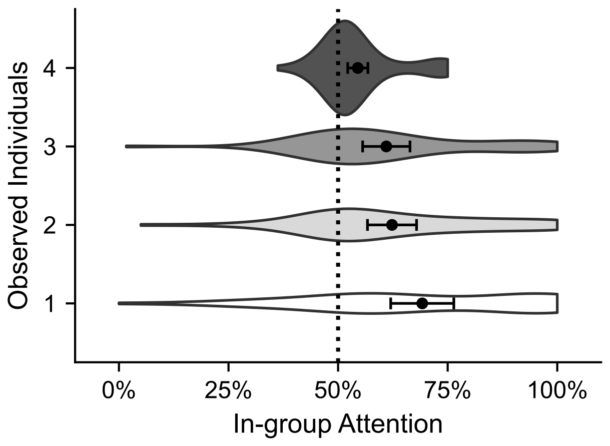

Figure S1: Frequency with which participants observed in-group versus out-group members as a function of the number of observed individuals (Experiment 2). Circles represent means, error bars represent 95% confidence intervals, and the height and width of the distributions indicate the density and range, respectively, of the data. The dotted line represents expected means in the absence of an intergroup bias.

observed it more often than those who rated it as less reliable. Nevertheless, participants exhibited an in-group attentional bias regardless of whether they rated the in-group as more reliable, $Z = 6.15$, $p < .001$, $OR = 4.97$, 95% CI = [2.98, 8.29]; equally reliable, $Z = 6.93$, $p < .001$, $OR = 2.43$, 95% CI = [1.89, 3.13]; less reliable, $Z = 2.22$, $p = .027$, $OR = 1.57$, 95% CI = [1.05, 2.34]; or were uncertain, $Z = 2.90$, $p = .004$, $OR = 2.32$, 95% CI = [1.31, 4.10].

Attention by Condition

The in-group attentional bias varied with the number of observed individuals (Figure S1), Wald $\chi^2(3) = 20.61$, $p < .001$, partial $R^2 = .04$, 90% CI = [.02, .07]. According to Bonferroni-corrected post hoc tests, participants who observed one individual per round were more likely to observe in-group members than those who observed four individuals. On average, participants observed in-group members 69% of the time (95% CI = [62%, 76%]) when observing one individual per round, $Z = 4.99$, $p < .001$, $OR = 5.03$, 95% CI = [2.67, 9.49]; 62% of the time (95% CI = [57%, 68%]) when observing two, $Z = 4.21$, $p < .001$, $OR = 2.73$, 95% CI = [1.71, 4.37]; 61% of the time (95% CI = [56%, 66%]) when observing three, $Z = 3.96$, $p < .001$, $OR = 2.44$, 95% CI = [1.57, 3.79]; and 55% of the time (95% CI = [52%, 57%]) when observing four, $Z = 3.78$, $p < .001$, $OR = 1.43$, 95% CI = [1.19, 1.73]. Like the in-group-copying bias, the in-group attentional bias grew with the scarcity of social information.

Changes in Perceived Warmth and Competence

In-group biases in perceived warmth and competence decreased over the course of the game—warmth: $t(215) = -2.15$, $p = .033$, $d = -0.15$, 95% CI = [−0.28, −0.01]; competence: $t(215) = -2.65$, $p = .009$, $d = -0.18$, 95% CI = [−0.31, −0.05]. However, the number of observed individuals had no effect on the extent to which these perceptual biases changed—warmth: Kruskal-Wallis $\chi^2(3) = 0.12$, $p = .989$, $\epsilon^2 = .00$, 90% CI = [.00, .00]; competence: Kruskal-Wallis $\chi^2(3) = 2.45$, $p = .485$, $\epsilon^2 = .01$, 90% CI = [.00, .03].

Ratings and Final Scores

Curiously, participants' final scores in experiment 1 varied with both their pre-game reliability ratings, $F(3, 320) = 3.58$, $p = .014$, $\eta^2 = .03$, 90% CI = [.00, .06]; and their post-game reliability ratings, $F(3, 320) = 3.86$, $p = .010$, $\eta^2 = .03$, 90% CI = [.00, .07]. According to Bonferroni-corrected post hoc tests, participants who rated the in-group as less reliable before the game earned fewer points than those who rated the groups as equally reliable. Similarly, participants who rated the in-group as less reliable after the game earned fewer points than those who rated the in-group as more reliable.

Participants' final scores in experiment 2 did not vary with their pre-game competence ratings of the in-group, $t(213) = 0.12$, $p = .901$, partial $R^2 = .00$, 90% CI = [.00, .01]; or the out-group, $t(213) = -0.75$, $p = .457$, partial $R^2 = .00$, 90% CI = [.00, .03]. Final scores also did not vary with participants' post-game competence ratings of the out-group, $t(213) = -0.95$, $p = .342$, partial $R^2 = .00$, 90% CI = [.00, .03]; though they did vary with their post-game ratings of the in-group, $t(213) = 2.13$, $p = .034$, partial $R^2 = .02$, 90% CI = [.00, .06]. In line with experiment 1, participants who rated the in-group as less competent after the game earned fewer points.

One possible explanation for the relationship between final scores and pre-game reliability ratings is that participants saw themselves as typical of their own group and relied on this heuristic in the absence of data (Masel, 2007). If pre-game ratings were partly self-evaluations (Kaznatcheev et al., 2014), then rating the in-group as less reliable may have expressed self-doubt. However, this does not explain why lower-scoring participants tended to rate the in-group as less reliable or competent after the game. One possibility is that, because most participants relied on the in-group to guide their behavior, those who performed poorly were particularly inclined to blame their own group.

Dynamics

While transmission chains often result in change over experimental generations, the in-group-copying bias did not change over time—experiment 1: Wald $\chi^2(1) = 0.79$, $p = .375$, partial $R^2 = .00$, 90% CI = [.00, .00]; experiment 2: Wald $\chi^2(1) = 0.70$, $p = .403$, partial $R^2 = .00$, 90% CI = [.00, .01]. The in-group attentional bias remained similarly stable—experiment 1: Wald

$\chi^2(1) = 0.05$, $p = .826$, partial $R^2 = .00$, 90% CI = [.00, .00]; experiment 2: Wald $\chi^2(1) = 0.69$, $p = .407$, partial $R^2 = .00$, 90% CI = [.00, .00].

Chains

Did participant behavior vary across chains? In experiment 1, chain membership had no effect on the in-group-copying bias, Wald $\chi^2(5) = 3.54$, $p = .617$, partial $R^2 = .00$, 90% CI = [.00, .00]; in-group attentional bias, Wald $\chi^2(5) = 3.93$, $p = .560$, partial $R^2 = .00$, 90% CI = [.00, .00]; amount of cultural divergence, $F(5, 318) = 1.07$, $p = .379$, $\eta^2 = .02$, 90% CI = [.00, .03]; or rate of cultural divergence, $F(5, 312) = 1.31$, $p = .259$, partial $\eta^2 = .02$, 90% CI = [.00, .04]. Because there was only one chain per condition in experiment 2, any variation across chains has already been captured as an effect of condition.

Individual Learning

Reliance on individual learning did not vary with the number of observed individuals, $F(3, 212) = 2.02$, $p = .113$, $\eta^2 = .03$, 90% CI = [.00, .06].

References

Chmielewski, M., & Kucker, S. C. (2020). An MTurk crisis? Shifts in data quality and the impact on study results. *Social Psychological and Personality Science*. https://doi.org/10.1177/1948550619875149

Coppock, A. (2019). Generalizing from survey experiments conducted on Mechanical Turk: A replication approach. *Political Science Research and Methods*, *7*(3), 613–628. https://doi.org/10.1017/psrm.2018.10

Dennis, S. A., Goodson, B. M., & Pearson, C. A. (2020). Online worker fraud and evolving threats to the integrity of MTurk data: A discussion of virtual private servers and the limitations of IP-based screening procedures. *Behavioral Research in Accounting*, *32*(1), 119–134. https://doi.org/10.2308/bria-18-044

Kaznatcheev, A., Montrey, M., & Shultz, T. R. (2014). Evolving useful delusions: Subjectively rational selfishness leads to objectively irrational cooperation. *Proceedings of the 36th Annual Meeting of the Cognitive Science Society*, 731–736.

Kennedy, R., Clifford, S., Burleigh, T., Waggoner, P. D., Jewell, R., & Winter, N. J. G. (2020). The shape of and solutions to the MTurk quality crisis. *Political Science Research and Methods*, *8*(4), 614–629. https://doi.org/10.1017/psrm.2020.6

Masel, J. (2007). A Bayesian model of quasi-magical thinking can explain observed cooperation in the public good game. *Journal of Economic Behavior & Organization*, *64*(2), 216–231. https://doi.org/10.1016/j.jebo.2005.07.003

McKelvey, R. D., & Zavoina, W. (1975). A statistical model for the analysis of ordinal level dependent variables. *The Journal of Mathematical Sociology*, *4*(1), 103–120. https://doi.org/10.1080/0022250X.1975.9989847

Veall, M. R., & Zimmermann, K. F. (1996). Pseudo-R^2 measures for some common limited dependent variable models. *Journal of Economic Surveys*, *10*(3), 241–259. https://doi.org/10.1111/j.1467-6419.1996.tb00013.x
